# Screening the human exome: a comparison of whole genome and whole transcriptome sequencing

**DOI:** 10.1186/gb-2010-11-5-r57

**Published:** 2010-05-28

**Authors:** Elizabeth T Cirulli, Abanish Singh, Kevin V Shianna, Dongliang Ge, Jason P Smith, Jessica M Maia, Erin L Heinzen, James J Goedert, David B Goldstein

**Affiliations:** 1 Center for Human Genome Variation, Duke University School of Medicine, Box 91009, Durham, NC 27708, USA; 2 Infections and Immunoepidemiology Branch, Division of Cancer Epidemiology and Genetics, US National Cancer Institutes of Health, 6120 Executive Boulevard, Rockville, MD 20852, USA

## Abstract

**Background:**

There is considerable interest in the development of methods to efficiently identify all coding variants present in large sample sets of humans. There are three approaches possible: whole-genome sequencing, whole-exome sequencing using exon capture methods, and RNA-Seq. While whole-genome sequencing is the most complete, it remains sufficiently expensive that cost effective alternatives are important.

**Results:**

Here we provide a systematic exploration of how well RNA-Seq can identify human coding variants by comparing variants identified through high coverage whole-genome sequencing to those identified by high coverage RNA-Seq in the same individual. This comparison allowed us to directly evaluate the sensitivity and specificity of RNA-Seq in identifying coding variants, and to evaluate how key parameters such as the degree of coverage and the expression levels of genes interact to influence performance. We find that although only 40% of exonic variants identified by whole genome sequencing were captured using RNA-Seq; this number rose to 81% when concentrating on genes known to be well-expressed in the source tissue. We also find that a high false positive rate can be problematic when working with RNA-Seq data, especially at higher levels of coverage.

**Conclusions:**

We conclude that as long as a tissue relevant to the trait under study is available and suitable quality control screens are implemented, RNA-Seq is a fast and inexpensive alternative approach for finding coding variants in genes with sufficiently high expression levels.

## Background

The study of common human diseases is rapidly moving away from an exclusive focus on common variants using genome-wide association studies and toward sequencing approaches that represent most variants, including those that are rare in the general population.

Although rapidly falling, the per base costs of next generation sequencing platforms still preclude the generation of large sample sizes of entirely sequenced genomes at high coverage. In addition to this economic constraint, it is widely appreciated that the very large number of variants identified in such studies will make it difficult to use association evidence alone to identify causal sites. For these reasons, there has been considerable interest in focusing attention on coding variants as a first step at complete representation of human variation. Part of the motivation for this approach stems from the experience with Mendelian diseases, in which 59% of the causal variants are either missense or nonsense mutations [1]. Although there has been considerable speculation on the topic, there are in fact no solid data showing that the picture is any different for common diseases, which may also be influenced by variants that are in or near protein coding sequence [1].

The most comprehensive approach for focusing on exons alone is clearly exome capture, where regions matching a defined set of coding exons are pulled from the genomic DNA (gDNA) using microarrays and then sequenced. However, this approach requires an initial and costly hybridization step. The cost of exome sequencing has contributed to the interest in sequencing the transcriptome (RNA-Seq) as an alternative, and possibly easier and less expensive strategy [2]. While this approach will clearly miss poorly expressed genes in whatever tissue is being studied, it does have the advantage of generating additional information, such as gene expression level and splicing patterns.

Although exome capture was demonstrated to identify approximately 95% of genomic single nucleotide variants (SNVs) in curated and non-paralogous exons [3], it is not currently known to what extent SNVs identified by RNA-Seq capture the full set of exonic SNVs identified by genomic sequencing. If the ability to capture SNVs by RNA-Seq is highly dependent on expression level, then this method would be useful only when performed in the appropriate tissue type. If, on the other hand, RNA-Seq at high coverage allows SNVs to be captured even in genes that are not highly expressed, then both methods could be useful for opening up sequencing studies to larger datasets in more diverse scientific studies.

Here, we have sequenced the entire genome and transcriptome of a single individual to high coverage. By comparing the SNVs identified in the transcriptome at different levels of coverage to those identified in the gDNA, we are able to directly evaluate how well RNA-Seq captures genomic variants.

## Results

### Alignment and coverage

Both DNA and RNA were extracted from peripheral blood mononuclear cells (PBMCs) from the same individual. Both the cDNA and gDNA were sequenced using the Illumina Genome Analyzer II. Sequencing of the gDNA produced 1,450 million reads, each 75 bp long. Ninety percent of these reads were aligned to the human reference genome by BWA [4], and after removing potential PCR duplicates, the remaining 980 million reads produced a coverage of at least 5× for 94% of the bases in the genome (gaps in reference genome excluded), and the mean coverage for these bases was 24×.

Sequencing of the cDNA produced 280 million reads, half 75 bp long and half 68 bp long. TopHat [5] was used to align these reads to the reference genome, with exons and splice junctions restricted to the 45,455 protein-coding transcripts annotated in Ensembl version 50. Sixty-nine percent of these reads gave unique alignments, aligning to exactly one location in the specified transcriptome. Reads aligning to more than one location were discarded. After removing potential PCR duplicates, the remaining 81 million reads produced a mean coverage of above 5× for 51% of exons: these exons had a median coverage of 51×.

### Single nucleotide variants and overlap between datasets

We used SAMtools to call SNVs in our aligned gDNA and cDNA sequences. Indels and large structural variants were not analyzed. SAMtools called 51,055 SNVs in protein-coding exons in gDNA and 64,128 in cDNA. Of these, 48,740 in gDNA and 40,605 in cDNA passed quality control filters, and 19,054 of these overlapped between the two datasets in terms of position. When considering overlap between cDNA and genomic SNV calls, two measures were examined: sensitivity and specificity. Sensitivity was defined as the number of true positives (SNVs overlapping between the two datasets) divided by the number of true positives plus the number of false negatives (SNVs existing in the gDNA but not the cDNA). Specificity was defined as the number of true positives divided by the number of true positives plus the number of false positives (SNVs existing in the cDNA but not the gDNA). Quality control filters were optimized to maximize both the sensitivity and specificity in this study. In this dataset the sensitivity was 0.39 and the specificity was 0.47. If an exact match of the genotype was required as well as location, then the sensitivity fell to 0.35 and specificity to 0.42.

SNVs called in the gDNA and cDNA were also compared with entries in dbSNP. It was found that 90% of the gDNA exonic SNVs corresponded to a dbSNP entry, while this was true of only 56% of the cDNA SNVs. However, a further breakdown revealed that 94% of the true positive cDNA SNVs corresponded to a dbSNP entry, while only 23% of the false positives did the same. The false negatives corresponded to dbSNP entries 89% of the time.

### SNV identification at different levels of expression and coverage

**Figure 1:**
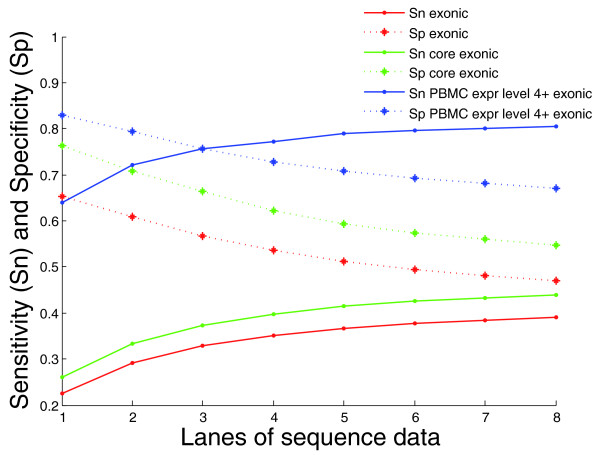
**Sensitivity and specificity as a function of the amount of sequence data generated**. Shown for all exons, core exons, and exons that are well expressed in PBMCs, designated as an expression level of at least 4% of the most highly expressed transcript in PBMCs. There were approximately 35 million sequence reads in each lane.

Many of the exons in Ensembl's transcript library are hypothetical and not confirmed to be expressed. A list of core exons as defined by the Affymetrix (Santa Clara, California, USA) Human Exon 1.0 ST Array was utilized to focus on exons with better curation. This list was further screened to only include exons present in Ensembl's list of canonical transcripts, resulting in 172,739 core exons. When focusing on just core exons, sensitivity and specificity rose to 0.44 and 0.55, respectively (Figure 1), which coincided with the percentage of exons having at least 5× coverage rising to 61% (median coverage for these exons was 57×).

**Figure 2:**
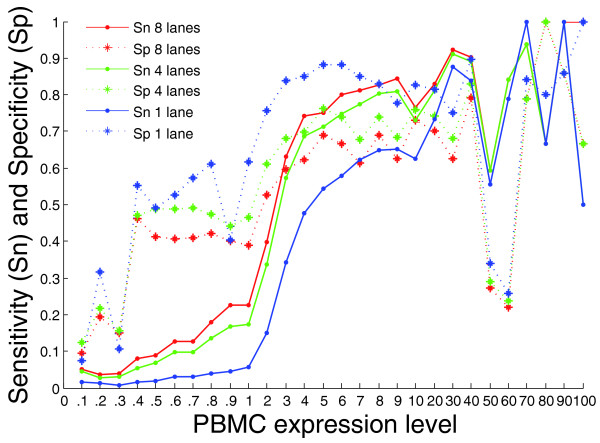
**Sensitivity and specificity by PBMC expression level**. The level of PBMC expression was broken up into bins based on a log scale. The expression value is written as the percent of the most highly expressed transcript in the dataset. The measures of sensitivity and specificity are shown for increasing levels of PBMC expression, for sequence data from one lane, four lanes and eight lanes. There were approximately 35 million sequence reads in each lane.

We then evaluated how the number of reads and the level of expression affected the specificity and sensitivity of cDNA sequencing. Using data from previous studies on the level to which most of the transcripts containing these core exons are expressed in PBMCs [6], we defined expression level for each transcript as a percentage of the most highly expressed transcript in that tissue. For exons in our dataset the sensitivity and specificity both rise as known PBMC expression increases, until an expression level of 4% of the most highly expressed transcript, at which point both measures asymptote, with variants called about equally well for all expression levels above this (Figure 2). Ninety-four percent of exons from genes above this expression level, or 'PBMC-expressed genes', had at least 5× coverage, and the median coverage for exons with at least 5× coverage was 126×. The sensitivity also rose to 0.81 and the specificity to 0.67.

**Figure 3:**
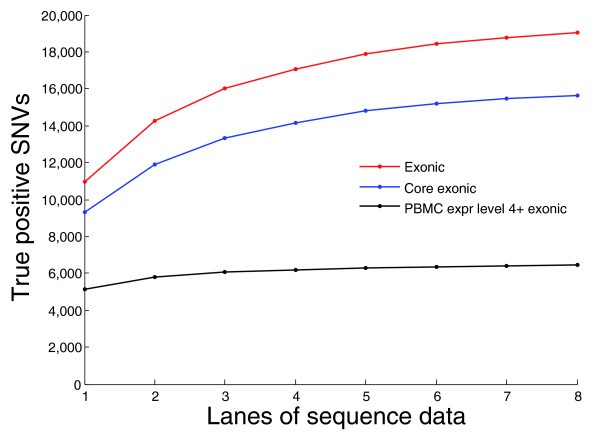
**True positive SNVs identified as a function of the amount of sequence data generated**. The number of true positive SNVs identified by RNA-Seq is shown for between one and eight lanes of sequence data, for exonic, core exonic and PBMC-expressed SNVs. PBMC-expressed genes are designated as those with an expression level of at least 4% of the most highly expressed PBMC transcript. There were approximately 35 million sequence reads in each lane.

**Figure 4:**
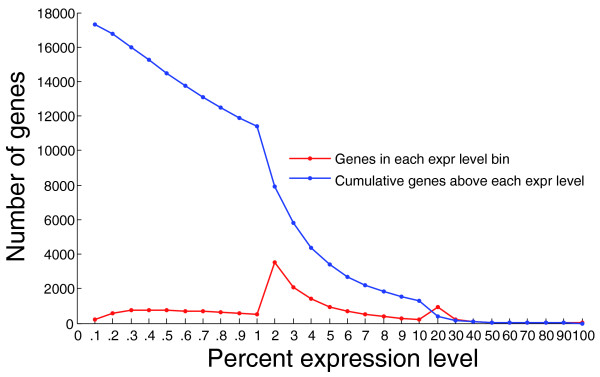
**Distribution of genes by PBMC expression level**. The number of genes lying within each PBMC expression level bin is shown in red. The cumulative number of genes expressed above each expression level is listed in blue. The expression value is written as the percent of the most highly expressed transcript in the dataset.

We also evaluated how the absolute number of true positive SNVs called depends on the amount of sequence data, in lanes, for all exons and for exons from PBMC-expressed genes (Figure 3). Seventy-nine percent of the 6,434 true positive variants identified in PBMC-expressed genes were identified with even one lane of sequence data, which is approximately 35 million reads in this instance. The total number of variants identified in all genes, however, increased substantially as more lanes were added. For the approximately 4,500 PBMC-expressed genes (Figure 4), even a single lane can be expected to capture most of the coding variants present.

We also found that the percent overlap with dbSNP changed as expression level and coverage changed. Although the percentage of SNV calls with a corresponding dbSNP entry remained relatively stable at all expression and coverage levels for the true positives and false negatives in our dataset, this was not true of the false positives. The percentage of false positives that overlapped with dbSNP decreased as coverage increased (Supplemental figure S3 in Additional file 1) and increased as expression level increased (Supplemental figure S4 in Additional file 1).

### SNV identification in genes with and without paralogs

An inspection of false positive SNVs identified in the cDNA revealed that some arose from alignment of a read to the wrong gene. In these cases the correct gene and the gene chosen for alignment always had very similar sequences. To determine if specificity would increase in a set of unrelated genes, SNVs were called separately for two groups of genes: those with paralogs, as annotated by Ensembl, and those without. When SNVs were restricted to exons in 12,124 transcripts from genes without annotated paralogs, the overall sensitivity rose from 0.39 to 0.42 and the specificity rose from 0.47 to 0.54. If restricted to PBMC-expressed genes without paralogs, the sensitivity actually dropped slightly from 0.81 to 0.80, but the specificity again rose from 0.67 to 0.72. In contrast, for SNVs in exons of 33,331 transcripts from genes with paralogs, the sensitivity was 0.38 and the specificity was 0.45 (sensitivity 0.81 and specificity 0.65 in PBMC-expressed genes with paralogs).

### Single nucleotide variant identification at different read depths

We studied the effect of read depth on specificity by examining the read depth at individual SNV calls in our complete RNA-Seq dataset of eight lanes. We found that at a read depth of 3 (the minimum required for a variant to be called), the specificity for SNVs found in the complete set of exons was only 0.28, but that as read depth increased, so did specificity, until it reached a plateau of between 0.6 and 0.75 for read depths between 50 and 1,200 (Supplemental figure S1 in Additional file 1). The 13,892 SNVs called between these read depth levels had a specificity of 0.67 (sensitivity of 0.19). However, above a depth of 1,200, the specificity fell again, becoming as low as 0.05 for the 94 SNVs with read depths greater than 2,000. Similar trends were found for core exonic SNVs (sensitivity 0.22 and specificity 0.77), SNVs in PBMC-expressed genes (sensitivity 0.59 and specificity 0.84), and SNVs in PBMC-expressed genes without paralogs (sensitivity 0.58 and specificity 0.90) when restricting to SNVs with a read depth between 50 and 1,200 (Supplemental figure S1 in Additional file 1).

We also studied the effect of read depth on specificity as the dataset increased from one to eight lanes. We found that at only one lane of RNA-Seq data, the specificity was 0.5 for SNVs with a read depth of 3, which was far better than the value of 0.28 found when using all eight lanes of data. Again, the specificity increased as the read depth increased, but in this lower coverage dataset the specificity reached a plateau of between 0.6 and 0.75 at greater than 10 reads, far earlier than the 50 reads needed for specificity stability in the eight lane dataset. The specificity also decayed at a much lower read depth in this smaller dataset, becoming less than 0.6 when the read depth was greater than 150. As the dataset increased from one lane to all eight lanes, the overall specificity continually decreased (Figure 1), and the minimum and maximum read depth value required for specificity to remain stable continually increased (Supplemental figure S2 in Additional file 1).

## Discussion

If one simply considers all coding SNVs, our study suggests that about 40% can be identified by RNA-Seq using PBMCs as the RNA source. If we focus, however, on only PBMC-expressed genes, we find that 81% of coding variants are identified. This suggests that RNA-Seq may be a workable alternative for identifying exonic variants when performed in the appropriate tissue for the trait of interest.

One limiting factor in variant identification by RNA-Seq is the ability to uniquely align a given read. Although we were able to align 78% of our reads to a location in the transcriptome, only 69% aligned to exactly one location and could be kept in the analysis. Because exons are some of the most conserved regions of the genome, without the help of intervening and variable intron sequences it is much harder to align a read to the correct gene, and especially to have it align to only one location. This limits one's ability to identify variants in certain genomic locations, lowering the sensitivity of this method. Furthermore, if a read is uniquely aligned to the wrong gene, such as a paralog, then this can result in false positive SNVs being called in the cDNA. Restricting SNV calls to genes without paralogs did increase specificity from 0.47 to 0.54 and sensitivity from 0.39 to 0.42.

Another limiting factor is coverage. Because genes are expressed at different levels, in the random sampling of transcripts that are sequenced there will be gross imbalances. Some transcripts will have more than 1,000-fold coverage while other transcripts, although also expressed to some level in that tissue, will have coverage that is too low for variants to be accurately called. Given the diminishing returns of additional sequencing in terms of variants called (Figure 3), it simply will not be possible to use RNA-Seq to capture all exonic variants in genes with low expression levels. RNA-Seq is most useful for identifying variants in a tissue type that highly expresses genes related to the trait under study. One interesting possibility to improve the proportion of SNVs that can be called would be to use more than one tissue type as a source for the RNA. For example, data available on expression of core exons in muscle shows that adding cDNA from this tissue to PBMC cDNA would increase the number of adequately expressed transcripts by 68% [6,7]. There are no expression data for a more easily accessible tissue such as skin for this exon array publicly available; however, one can extrapolate that adding cDNA from almost any tissue would be similarly beneficial to analysis.

A large number of false positive SNVs were identified in this dataset: even when restricted to PBMC-expressed genes without paralogs the specificity was only 0.72. Many of these false positives, however, were due to the very high coverage produced in this study. At low levels of coverage, reads that could produce false positive calls are not yet abundant enough to pass the quality control filters used, and the specificity remains high. However, as more reads are added to the dataset, the number of incorrect alignments and sequencing mistakes increases, pushing more and more of these false positive calls over the quality control filters. Thus, the more coverage that is added in the search for true positives, the more false positives that will appear in the data. We found that the specificity for the dataset as a whole could be increased from 0.47 to 0.67 (and from 0.72 to 0.9 for PBMC-expressed genes without paralogs), at a substantial cost to sensitivity, by restricting the permissible read depth range for SNV calls. The specificity was low at low read depths, as is expected when a call is supported by less data; interestingly, the minimum read depth required for high specificity increased as coverage increased, supporting the view that high coverage introduces more noise and comes with a requirement for stricter quality control. An additional support to this view was the finding of low specificity at very high read depths. Both a minimum and a maximum read depth cutoff are advisable for increasing the specificity.

Some of the false positives found in this dataset are certainly due to the incorrect alignment of reads, as even when a gene has no annotated paralog it can have sections with sequence similarities to other genes that permit errors during alignment. Another alignment problem that is unique to RNA-Seq is incorrect alignment at the very ends of a read due to splicing. Because reads that span exons require a certain number (four in our study) of bases to fall on both sides of the exon-exon boundary for proper alignment, reads that cross an exon boundary at the very edge of the read will have between one and three bases aligned to the wrong location. Our study removed most of this type of false positive SNV during the quality control process. Another scenario that would produce a seemingly false positive SNV would be sufficient coverage in the cDNA to call the variant but insufficient coverage in the gDNA sequencing to do the same: however, in our study such bases had a median coverage of 27× in the gDNA, which does not support this explanation. Also, previous studies have shown that variants can be present in the RNA but not the gDNA due to RNA editing [2,8].

There are also likely to be disagreements between gDNA and cDNA sequencing stemming from expression differences. For example, some SNVs found in the genomic sequence may be missed in the cDNA due to expression balances, when an individual is heterozygous for a given SNV yet the reference allele is much more highly expressed. Also, an SNV may be called heterozygous in the gDNA but homozygous in the cDNA due to expression imbalances. In our dataset, 10% of the 19,054 SNVs that overlapped by location between gDNA and cDNA were heterozygous in gDNA and yet homozygous in cDNA. Differences in zygosity between the two methods can also result from insufficient coverage in one dataset or the other: for example, 1% of these 19,054 SNVs were homozygous in gDNA and yet heterozygous in cDNA, and the median coverage for these SNVs in gDNA was only 12×, compared to 28× for SNVs that matched for zygosity. It is likely that many of the discrepancies where the SNV was heterozygous in gDNA and homozygous in cDNA also resulted from low coverage, as the median coverage for this group was only 8× in the cDNA. It should also be noted that only 13 of the 19,054 SNVs that overlapped by location had completely mismatched alleles, such as the cDNA being homozygous for a G and the gDNA homozygous for C when the reference allele was A.

Our study found that although the percent overlap of our false positive SNVs with dbSNP entries was far less than that of true positive (94%), false negative (89%), or all exonic gDNA (90%) SNVs, it was still substantially greater than zero (23%). Furthermore, we found that the percent of false positives corresponding to dbSNP entries increased as the PBMC expression level increased (Supplemental figure S4 in Additional file 1), implying that the 'false positives' seen at high expression levels may actually be true positives that were simply not seen in the gDNA due to issues with coverage or alignment or because of RNA editing. Additionally, we found that only 13% of the false positive SNVs found in dbSNP had been genotyped in HapMap, compared with 69% of the true positives found in dbSNP. This suggests that many of the dbSNP entries matching false positives are less well-curated, with less supporting evidence. A brief inspection of a subset of the SNVs showed that false positives were more likely to have cDNA evidence (as opposed to gDNA evidence) supporting their dbSNP entry than were true positives; this could be a reflection of either misalignment of the cDNA reads for dbSNP entries, or RNA edits that would not be seen in the gDNA. Finally, we found that the percent overlap of false positives with dbSNP decreased as the number of lanes of sequence data increased (Supplemental figure S3 in Additional file 1). This finding corresponds with the fact that the overall specificity decreased as coverage increased (Figure 1); these phenomena are likely caused by the increasing ability of the random noise inherently present in the data to overcome quality control cutoffs as more and more reads were added, as described above.

Two previous studies have also looked at using RNA-Seq to identify SNVs. Chepelev *et al*. [2] used a dataset of 27 million uniquely aligned 30-bp RNA-Seq reads; while they detected 50% of known exons with at least 1× coverage, and identified approximately 11,000 SNVs, they did not compare these data to gDNA sequence and thus could not calculate sensitivity or specificity of their methods. Shah *et al*. [8] used a dataset of 183 million RNA-Seq reads of 38.9 bp each, 55 million of which aligned to exons or exon junctions. They compared these data to 2.5 billion aligned gDNA reads of 48.2 bp to better understand the evolution of mutation in a lobular breast tumor. Although they showed that the number of SNVs called through RNA-Seq increased as the number of reads increased, they did not discuss the sensitivity or specificity of SNV calls when compared to whole genome sequencing, nor did they analyze how the number of SNVs changed as known expression level changed.

Another technology that has been used to sequence coding variants is exome capture. Because this method sequences reads that are from gDNA but enriched for the portions of interest, there are no complications with aligning splice junctions or being limited by expression level. A recent study showed that when restricted to the non-paralogous exons of 16,496 curated protein coding genes, exome capture utilizing 41 million 76-bp reads (one quarter the number of bases we aligned) captured SNVs with a sensitivity of 95% and a specificity of 90% [3]. Although this performance outshines the RNA-Seq data presented here, RNA-Seq does have some advantages beyond the ability to inexpensively genotype exonic SNVs. It can also be used to identify expression differences between individuals or even between alleles within an individual, which can lead to discovery of a nearby causal variant even if it is not exonic. RNA-Seq may also provide insight into novel exons, splice junctions or splice forms in the tissue or cell type being studied that might not be recognized as protein coding in genomic sequencing, or captured with targeted exomic sequencing.

While it is most useful to perform RNA-Seq in a tissue relevant to the trait under study, other tissues can also be of some use. For example, data from Heinzen *et al*. [6] revealed an r^2 ^of 0.23 between transcript expression in PBMCs and in brain, and that 63% of the transcripts highly expressed in brain were also expressed in blood to a level that allowed for consistent SNV detection by RNA-Seq (defined as expression level of at least 4% of the most highly expressed transcript in both).

## Conclusions

Here we show that RNA-Seq captured 81% of the exonic variants from genes that were well expressed in the source tissue. Although its usefulness is limited to these genes, the cheaper cost involved, as well as the extra information gained about expression and splice variants, may make this method a workable alternative to genomic sequencing or exome capture for groups that have access to the right types of tissue.

## Materials and methods

### Sample preparation and sequencing

DNA was extracted from PBMCs using the QIAGEN Autopure LS (Venlo, The Netherlands). RNA was extracted from viable PBMCs using the Qiagen RNeasy kit. The DNA was prepared for sequencing according to Illumina's gDNA sample prep kit protocol: randomly fragment the DNA by nebulization, end repair, add a single A base, adaptor ligation, run a gel to isolate 300-bp fragments, and PCR amplification. The total RNA was prepared according to the Illumina RNA seq protocol: briefly, globin reduction, polyA enrichment, chemical fragmentation of the polyA RNA, cDNA synthesis, and size selection of 200-bp cDNA products. Next, the size-selected libraries were used for cluster generation on the flow cell. All prepared flow cells were run on the Genome Analyzer II using the paired-end module: nine flow cells (with eight lanes each) for the gDNA and one flow cell for the cDNA. The paired-end reads for the gDNA were each 75 bp long, although one flow cell only produced single reads of 75 bp each. Due to a machine error near the end of the read 1, the paired cDNA reads were not able to be matched to each other and read 1 was only 68 bp long while read 2 was 75 bp. The reads are available in the NCBI Sequence Read Archive [9], under study ID SRP001691. The Illumina GA Pipeline version was 1.4.0. This pipeline produced the quality score for each nucleotide in standard Illumina format where the base was 64 (Illumina quality score = Qphred +64, where Qphred = -10log10(e) and e = estimated probability of a nucleotide being wrong). We converted the quality scores for each nucleotide to standard Sanger fastq format, where the base was 33.

### Alignment and single nucleotide variant identification

gDNA was aligned to the reference genome (NCBI build 36 Ensembl release 50) using the BWA software (version 0.4.9) [4]. cDNA was aligned to the reference genome using TopHat [5]. The -GFF function utilized a transcript library downloaded from Ensembl to specify known protein-coding transcripts and splice junctions, and the library was screened to remove contigs and mitochondrial DNA. The --no-novel-juncs option was used to restrict alignment to those exons and splice junctions included in this transcript library. To assist in alignment to small exons, the 75-bp reads were broken down into three 25-bp segments (68-bp reads into two 34-bp segments), which were then joined back together after being individually aligned. Two mismatches were permitted per 25-bp (or 34-bp) segment, and no mismatches were permitted in the 4-bp anchor region on either side of a splice junction. Introns were permitted to range in size from 10 bp to 500 kb. Only unique alignments were kept: that is, reads that aligned to exactly one location. Reads mapping to multiple locations were excluded using in-house software.

SAMtools (version 0.1.5c) was used to remove potential PCR duplicates via the rmdup (paired reads) and rmdupse (single reads) command [10]. It was also used for SNV identification, using the pileup command with the -c option and default settings. The SNVs were then filtered using SAMtool's variation filter with the default settings but removing the filter for a maximum allowed coverage per variant by setting it to 10 million for gDNA and 1 million for cDNA. SNVs lying outside exons as defined by the transcript library were removed. Indels were not considered. All SNVs were further screened for quality by only keeping those above a minimum SNP quality score: 30 for cDNA and 20 for gDNA. This score is calculated by SAMtools and is the Phred-scaled probability that the base at that location is identical to reference, with higher scores being less likely to be reference. SNVs were also excluded if there were fewer than three reads supporting the non-reference allele. cDNA SNVs were further screened to exclude all SNVs where more than 20% of the reads supporting the non-reference allele were from the first or last base of a sequence read.

### Coverage

Coverage of cDNA sequencing for each exon was calculating using in-house software. For each exon in the transcript library, coverage was calculated as the average number of reads covering each base within that exon.

### Paralogous genes

Genes were designated as paralogous using ENSG IDs as input in Genecards' paralog finder [11,12]. The list of paralogs from Ensembl was utilized, and genes were split into two groups: those with paralogs and those without. The 42 ENSG IDs not recognized by Genecards were individually examined for paralog status using Ensembl directly.

## Abbreviations

bp: base pair; gDNA: genomic DNA; PBMC: peripheral blood mononuclear cell; SNV: single nucleotide variant.

## Authors' contributions

ETC participated in the design of the study, performed analyses, and drafted the paper. AS performed analyses and processed the cDNA reads. KVS supervised the sequencing of gDNA and cDNA. DG performed analyses and processed the gDNA reads. JPS sequenced the gDNA and cDNA. JMM performed analyses and processed the gDNA reads. ELH provided expression data. JJG collected the cohort, prepared the samples, and reviewed and edited the paper. DBG designed and supervised the study and helped to write the paper. All authors read and approved the final manuscript.

## Supplementary Material

Additional file 1 **Supplemental figures S1 to S4**. Showing the specificity at different read depth levels and the overlap with dbSNP entries at different coverage levels and different expression levels. Click here for file
