# Supplementary material for: Screening the human exome: a comparison of whole genome and whole transcriptome sequencing
Source: Genome Biol. 2010 May 28;11(5):R57. doi: 10.1186/gb-2010-11-5-r57 (PMC2898068; doi:10.1186/gb-2010-11-5-r57)

Supplemental Figure 1. Specificity at different read depth levels. The data is from (A) all exonic, (B) core exonic, (C) PBMC-expressed, and (D) PBMC-expressed without paralogs RNA-Seq SNVs. The read depth is broken into bins: 3-10, 10-20, 20-30, …, 190-200, 200-300, 300-400, …, 900-1000, 1000-1200, 1200-1400, …, 1800-2000, and 2000-3151 (for B and C, the last bin is 2000-2949, and for D, the last bin is 2000-2236).

A: All exonic B: Core exonic

C: PBMC-expressed D: PBMC-expressed without paralogs

Supplemental Figure 2. Specificity at different read depth levels as the lanes of sequence data increase. The data is from (A) 1 lane of sequence data, (B) 2 lanes of sequence data, …, and (H) 8 lanes of sequence data. The read depth is broken into bins: 3-10, 10-20, 20-30, …, 190-200, 200-300, 300-400, …, 900-1000, 1000-1200, 1200-1400, …, and 1800-2000 (for F, the last bin is 2000-2500, for G it is 2000-2753, and for H it is 2000-3151). As the number of lanes increases, the specificity of the SNVs in the lowest read depth bins decreases.

A: 1 lane B: 2 lanes

C: 3 lanes D: 4 lanes

E: 5 lanes F: 6 lanes

G: 7 lanes H: 8 lanes

Supplemental Figure 3. Overlap with dbSNP entries at different coverage levels. Shown are the true positives (found in both cDNA and gDNA), the false negatives (found in gDNA but not cDNA) and the false positives (found in cDNA but not gDNA) as the number of lanes of sequence data increases from one to eight.


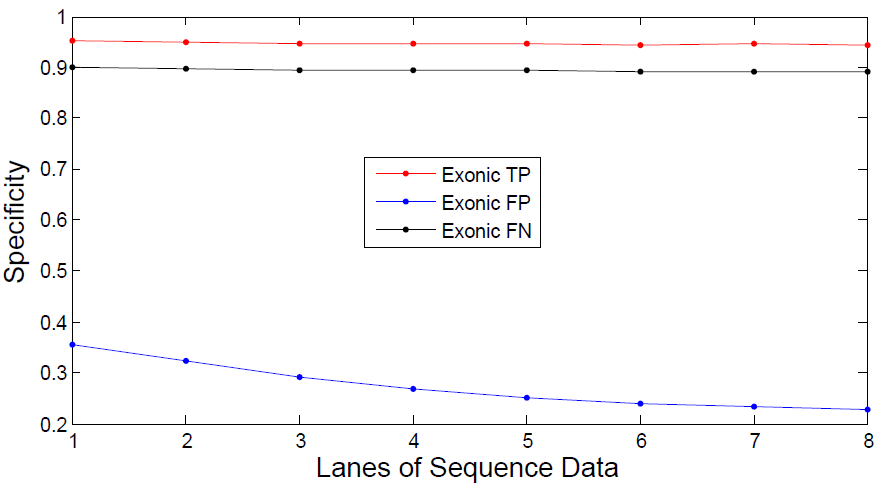


Supplemental Figure 4. Overlap with dbSNP entries at different PBMC expression levels. Shown are the true positives (found in both cDNA and gDNA), the false negatives (found in gDNA but not cDNA) and the false positives (found in cDNA but not gDNA) as the known level of PBMC expression increases from 0.1% to 100% of the most highly expressed PBMC transcript. The specificity becomes more variable at higher expression levels because the sample size decreases.


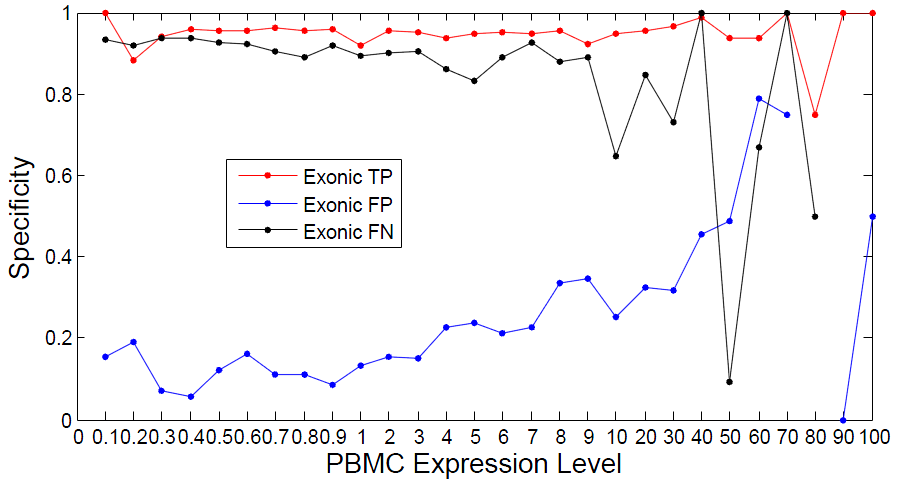

Supplement: Additional file 1 — Supplemental figures S1 to S4. Showing the specificity at different read depth levels and the overlap with dbSNP entries at different coverage levels and different expression levels. [file gb-2010-11-5-r57-S1.DOC]
